# Periodic Stratification of Colloids in a Liquid Phase Produced by a Precipitation Reaction and Gel Swelling

**DOI:** 10.1021/acs.langmuir.4c00533

**Published:** 2024-05-17

**Authors:** Pedram Tootoonchian, Gábor Holló, Rana Uzunlar, Istvan Lagzi, Bilge Baytekin

**Affiliations:** † Chemistry Department, Bilkent University, Ankara 06800, Turkey; ‡ Department of Physics, Institute of Physics, Budapest University of Technology and Economics, Budapest H-1111, Hungary; § HU-REN−BME Condensed Matter Physics Research Group, Budapest University of Technology and Economics, Budapest H-1111, Hungary; ∥ UNAM National Nanotechnology Research Center, Bilkent University, Ankara 06800, Turkey

## Abstract

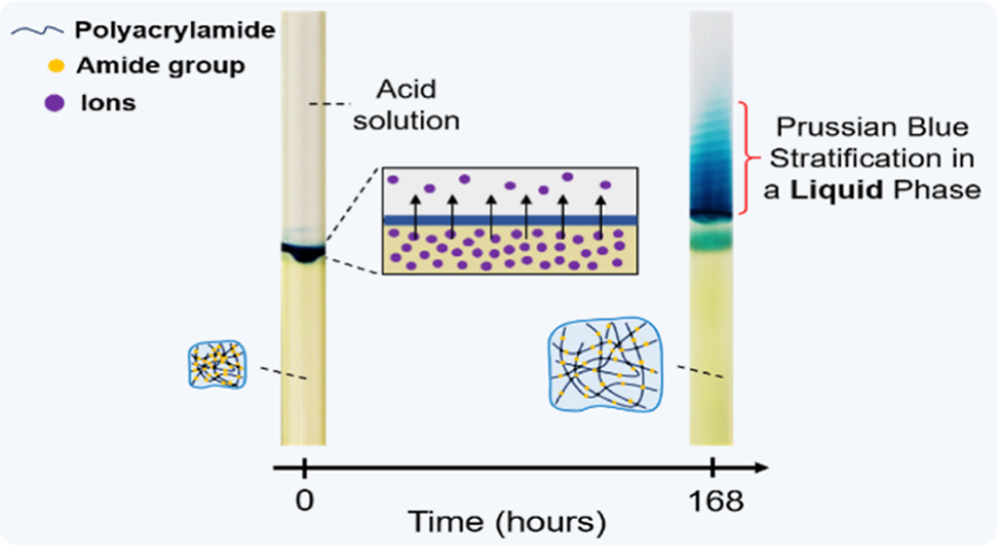

Pattern formation is a frequent phenomenon occurring in animate and inanimate systems. The interplay between the mass transport of the chemical species and the underlying chemical reaction networks generates most patterns in chemical systems. Periodic precipitation is an emblematic example of reaction–diffusion patterns, in which the process generates a spatial periodic structure in porous media. Here, we use the dormant reagent method to produce colloidal particles of Prussian blue (PB) and PB analogues at the liquid–gel interface. The generated particles produced a stable periodic stratification pattern in time in the liquid phase placed on top of the solid hydrogel. The phenomenon is governed by periodic swelling of the gel driven by the osmotic stress and stability of the formed particles. To illustrate the phenomenon, we developed an extended reaction–diffusion model, which incorporated the gel swelling and sedimentation effect of the formed colloids and could qualitatively reproduce the pattern formation in the liquid phase.

## Introduction

**Figure 1:**
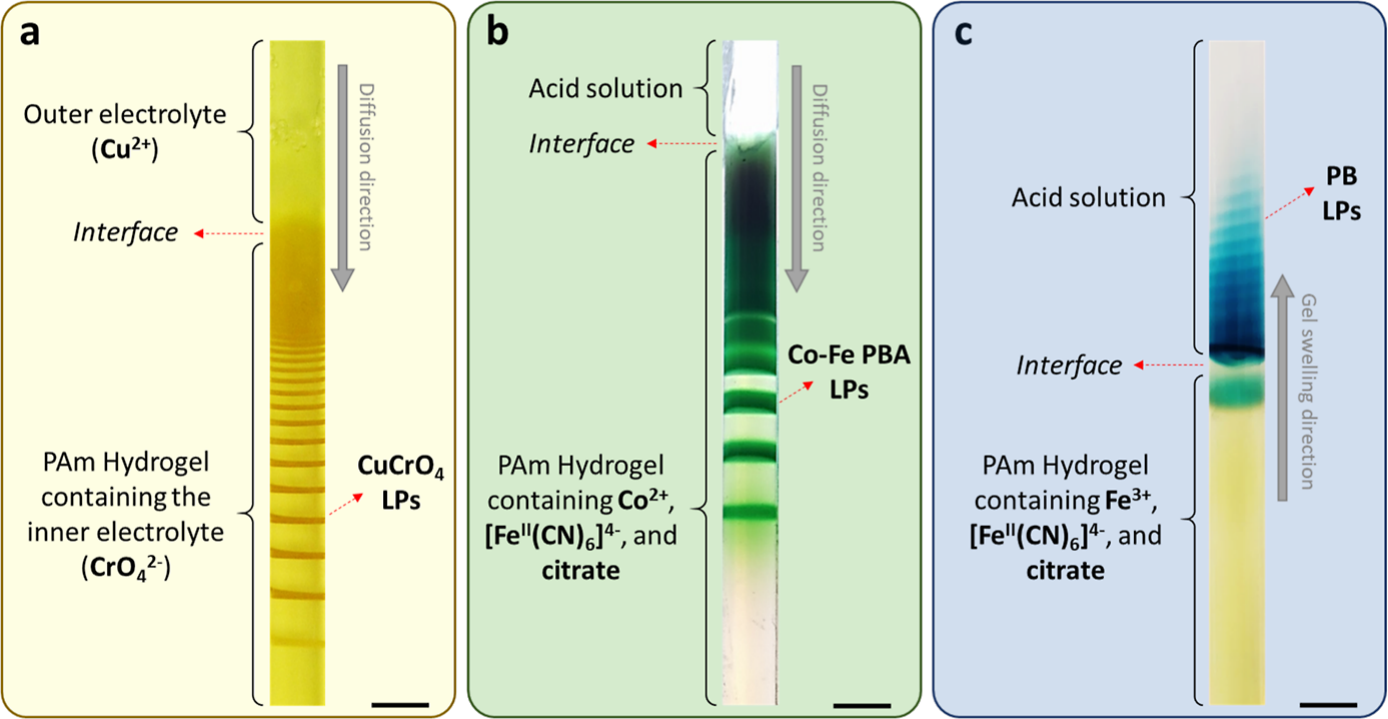
(a) Classical setup of periodic precipitation of CuCrO_4_ in a hydrogel medium. (b) Previously reported DR method for the formation of cobalt(II) ferrocyanide in the hydrogel medium. (c) Periodic stratification of PB colloids in the acid solution above a gel column using the DR method at *t* = 168 h. [Fe^3+^] = 0.01 M, [[Fe^II^(CN)_6_]^4–^] = 0.01 M, and [sodium citrate] = 0.3 M in the hydrogel were subjected to 0.6 M HNO_3_ (in 3:1 v/v % water/DMF mixture). The scale bar represents 5 mm.

Periodic precipitation (or Liesegang phenomenon) is one of the oldest self-organized structures discovered in reaction–diffusion systems.^1−6^ In these systems, pattern formation emerges due to the coupling of diffusion of chemical species to chemical reactions among the involved chemical compounds. Periodic precipitation manifests as distinct and regularly spaced precipitation bands,^7−10^ rings,^11,12^ or shells^13^ that emerge within a solid hydrogel or other porous medium (e.g., aerogel)^14^ due to the diffusion and reaction between chemical species that are initially spatially separated. In the classical (the most dominantly used) setup, one electrolyte is homogeneously distributed in a gel matrix (called the inner electrolyte). In contrast, the other electrode (called the outer electrolyte) is placed on top of the solid gel. The diffusion flux of the outer electrolyte drives the pattern generated in the gel (Figure 1a).^4^ Periodic precipitation has been primarily studied in chemical systems, and its principles have found applications in diverse fields, ranging from geology^15−17^ and biology^18,19^ to materials science.^20−24^ Periodic precipitation is a reaction–diffusion system characterized by a set of partial differential equations in its mathematical model. The mathematical models for periodic precipitation can vary depending on how they treat the precipitation process. There are two types of kinetic models that describe precipitation: the prenucleation model and the postnucleation model. In prenucleation models, nucleation occurs only if the product of the local concentrations of the reagents exceeds a threshold, such as the solubility product. On the other hand, in postnucleation models, nucleation continuously emerges in space, and pattern formation arises from thermodynamic instability or competitive particle growth.^4^

In our previous study, we introduced a new concept to generate periodic precipitation of Prussian blue (PB) analogues (PBAs) [cobalt(II) and copper(II) ferrocyanides] in a gel medium using the so-called dormant reagent (DR) method (Figures 1b and S1). In this approach, both electrolytes are homogeneously distributed in the gel. However, the metal cations are reversibly bonded to citrate, creating a complex (DR) that cannot react with potassium ferrocyanide to produce a precipitate. Once a strong acid diffuses into the gel (placed on top of the gel column), the acidic environment shifts the complexation to liberate free cations that react with ferrocyanide, generating periodic precipitation in the gel.^25^

This work investigates the precipitation reaction of PB and PBAs in a solid gel matrix using the DR method.^25^ PB and PBAs are widely used chemical compounds to create precipitation structures in a gel matrix.^26−31^ In contrast to the previous studies conducted on periodic precipitation in solid gels, we observed a striking phenomenon, namely, the formation of periodic stratification of colloids in an aqueous phase above the solid hydrogel column due to the periodic swelling of the gel and long-term stability of the colloids in the aqueous phase (Figure 1c).

## Experimental Methods

### Chemicals

Acrylamide (AA) (Sigma-Aldrich, 98% purity), *N*,*N*′-methylene(bis)acrylamide (BIS) (Sigma-Aldrich, 99% purity), potassium peroxydisulfate (KPS) (Sigma-Aldrich, 99% purity), *N*,*N*,*N*′,*N*′-tetramethylethylene-diamine (TEMED) (Sigma-Aldrich, 99% purity), iron(III) chloride hexahydrate (Sigma-Aldrich, 97% purity), indium(III) chloride (Sigma-Aldrich, 98% purity), silver(III) chloride hexahydrate (Sigma-Aldrich, 97% purity), potassium ferrocyanide trihydrate (AFG Bioscience), trisodium citrate dihydrate (Sigma-Aldrich), nitric acid (Sigma-Aldrich, 65%), and dimethylformamide (Carlo Erba Reagents) were used. All the aqueous solutions were prepared using ultrapure (type 1) water.

### Preparation of the Gels

The degree of cross-linking, the concentrations of the electrolytes and trisodium citrate, and the type and concentration of the acid (the activator) had to be optimized for pattern formation. For the gel solution, 0.73 g of AA, 0.01 g of KPS, and 0.003 g of BIS were dissolved in deionized water (3.5 mL). 1.5 mL of 1.0 M sodium citrate was added to the solution to make 0.30 M citrate in the pregel solution (in total 5 mL solution), and the mixture was stirred by ultrasonication for 1 min. Appropriate amounts of FeCl_3_·6H_2_O and K_4_[Fe^II^(CN)_6_]·3H_2_O were subsequently added to the solution to have 0.01 M of both Fe^3+^ and [Fe^II^(CN)_6_]^4–^ and then 50 μL of TEMED was added for gelation. The mixture was then transferred to a glass tube (with 100 mm length and inner diameter of 5.0 mm) using a 5.0 mL syringe to fill two-thirds of it and allowed to stay for 24 h for complete gelation. Finally, a 0.60 M HNO_3_ (containing 25% v/v DMF) solution is introduced to the top of the gel to fill the rest of the tube, and it is sealed with Parafilm. The reason for using DMF in the acid solution is the faster band formation, presumably due to the lower solubility of ferrocyanide ions in DMF compared to other ions [such as Fe(II), Fe(III), and citrate]. In this way, 0.69 M more ions were in the gel medium than in the aqueous phase (for the detailed calculation, see Supporting Information). All the experiments were carried out at room temperature (23 ± 0.5 °C).

### Characterization of the PB

We performed Raman spectroscopy, Fourier-transform infrared spectroscopy (FTIR), and scanning electron microscopy–energy-dispersive X-ray spectroscopy (SEM–EDX) measurements to characterize the PB samples.

The samples for Raman spectral analysis and SEM–EDX were prepared by collecting and combining the PB-colloid-containing liquid part (solution) over a minimum of 10 gel columns. The combined liquids were centrifuged twice at 10,000 rpm for 30 min, each time most of the supernatant was decanted, and the precipitate was washed with water and left to dry. The Raman spectra were obtained by using a WITec alpha300 SNOM-Raman confocal microscope. The SEM and EDX analyses were performed with an FEI Quanta 200 FEG model scanning electron micrcoscope with an accelerating voltage of 20 kV. Samples were coated with Au–Pd.

The KBr samples for FTIR analyses were prepared by placing drops of the PB-colloid-containing liquid on the gel columns between two KBr pellets. The transmittance spectra of DMF and HNO_3_ were also obtained through identical sample preparation and measurement. The spectra were obtained by using a Bruker Tensor 27 FTIR spectrometer.

For the dynamic light scattering (DLS) and zeta potential measurements, the solution above the gel of ten samples was collected and diluted, and the data were obtained at room temperature using a Malvern Zetasizer Nano ZS instrument with a DTS1070 cuvette. Each batch was measured three times, and 100 scans were used for each measurement.

## Results and Discussion

In a typical experiment, the PB (Fe^III^_4_[Fe^II^(CN)_6_]_3_) was generated by the reaction of iron(III) chloride with potassium ferrocyanide using the DR method discussed in the introduction (Figure 1c). One component of the precipitate [Fe(III) ions] was bonded to citrate anions (deactivator) to hinder the reaction of the iron ions with the potassium ferrocyanide.^32−34^ The formed citrate complex (the DR) can release free Fe(III) cations by reaction with a strong inorganic acid (the activator) layered on top of the gel. In the previous work using cobalt and copper ions, we presented that these ions reacting with ferrocyanide can generate periodic precipitation of PBAs in the solid hydrogel (Figure 1b). Interestingly, using iron(III) instead of cobalt(II) and copper(II) ions, we observed no pattern formation in the gel but found a peculiar phenomenon, namely, the periodic stratification of PB colloids above the gel in the liquid phase, which was stable over several days (Figure 1c and Movie S1).

The preparations of the PB systems using different experimental parameters showed that periodic stratification of PB colloids in the liquid phase above the gel was observed for samples with the following common features. The first feature is the formation of fine PB particles in the gel–liquid interface when the acidic solution was layered on top of the solid gel column. These particles could not penetrate the gel matrix and accumulated at the gel–liquid interface. During the time we waited for the formation of the patterns of PB in the gel, the gel started to swell. This swelling can be attributed to the osmotic concentration difference between the liquid and gel phases, since the concentration of ions in the gel was higher than that of the acid solution. In the beginning, the gel column could not swell continuously because the static friction force between the gel column and the inner surface of the test tube was compensated for by the increasing force due to swelling. Once the swelling force overcame the static friction, the gel column instantaneously expanded, increasing its volume. This phenomenon repeated periodically resulted in “ejection” and periodic stratification of the fine PB particles formed at the liquid–gel interface. It should be noted that another essential ingredient of the pattern formation in the liquid phase is the generation rate of the free cations in the gel from the complex induced by the diffusion front of the acid. A slow production rate of the cations and diffusion contribute to the pattern formation in solution rather than the Liesegang pattern in the gel.

**Figure 2:**
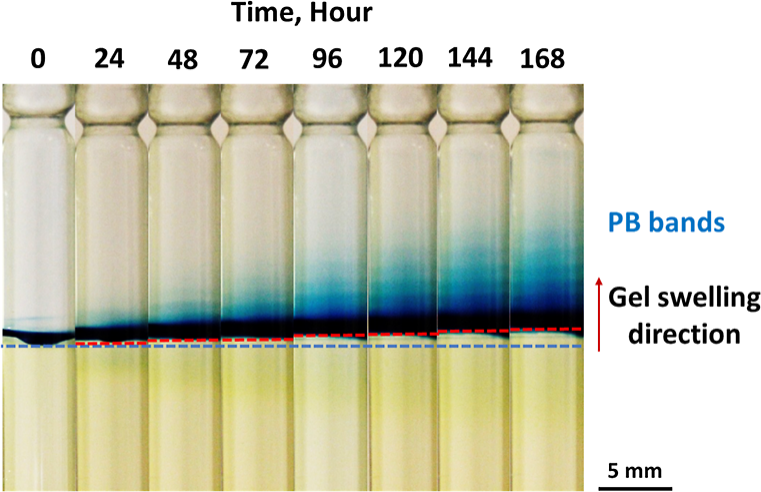
Spatiotemporal evolution of the stratification of PB colloids in the acid solution above the gel column due to the periodic swelling of the gel. [Fe^3+^] = 0.01 M, [[Fe^II^(CN)_6_]^4–^] = 0.01 M, and [sodium citrate] = 0.3 M in the hydrogel were subjected to 0.6 M HNO_3_ (in 3:1 v/v water/DMF). The dashed blue and red lines indicate the positions of the initial and actual gel surfaces, respectively. The diameter of the glass tube was 5 mm.

Figure 2 and Movie S1 show evidence of the swelling of the gel column over time and the formation of the stratified layers of PB colloids. The formation of the layers in the liquid phase evolved linearly over time (Figure S2a), indicating periodic swelling of the gel column with a constant time period. This observation differs from the band formation in periodic precipitation in gels. In this case, the correlation between the time of the band formation and the corresponding band number is nonlinear. More specifically, the distance of the precipitation bands measured from the liquid–gel interface is linearly proportional to the square root of time, indicating that this pattern formation is governed by diffusion.^4^Figure S2b shows the position of each layer (indicated as the middle of the layer) measured from the liquid–gel interface as a function of band number. The distance between two consecutive stratified layers increased over time, resembling the periodic precipitation behavior.^4^ We observed that the gel slightly darkened over time. It could be due to the unreacted iron(III) and ferrocyanide ions. When the acid solution was added onto the top of the gel, some unreacted ferrocyanide ([Fe(CN)_6_]^4–^) ions diffused into the solution phase and reacted with nitric acid to form ferricyanide ([Fe(CN)_6_]^3–^), which has a yellow/orange color at low concentrations. In addition, the released iron(III) ions from the citrate complex have a yellow/brown color. Therefore, the color of the gel turned darker yellow after some time.

**Figure 3:**
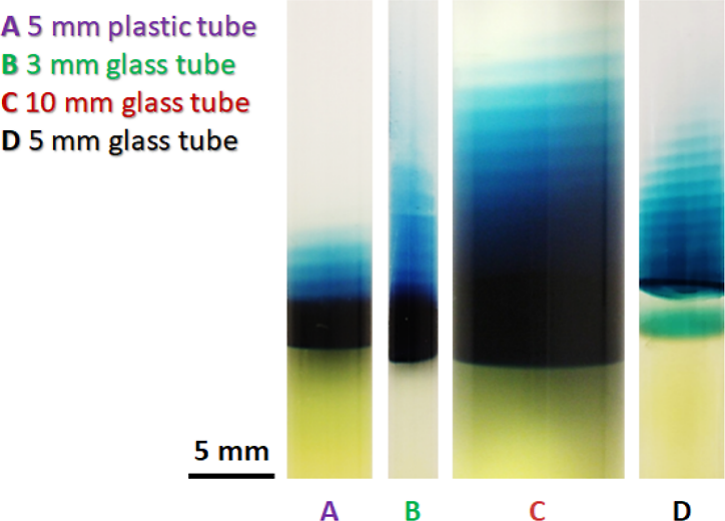
Effect of the chemical composition and diameter of the test tubes on the periodic stratification of the PB colloids in the acid solution above the gel column due to the periodic swelling of the gel at *t* = 168 h. [Fe^3+^] = 0.01 M, [[Fe^II^(CN)_6_]^4–^] = 0.01 M, and [sodium citrate] = 0.3 M in the hydrogel were subjected to 0.6 M HNO_3_ (in 3:1 v/v water/DMF).

The periodic stratification can be observed in glass test tubes with various diameters and plastic test tubes as well, highlighting that this phenomenon is not only attributed to the glass surface (Figure 3 and Movie S2). We plotted the position of the stratified layers (measured from the liquid–gel interface) formed in other setups (plastic tube with a diameter of 5 mm and glass tube with a diameter of 3 mm, Figure S3). These graphs show similar trends in the increase of positions of the layers and the distance between the consecutive stratified layers as a function of the layer number (in all three cases, the position of the fifth layer is ∼6 mm measured from the liquid–gel interface and the gap between the fourth and fifth layers is ∼1 mm). This indicates that the types of tubes and their diameter have no significant effect on the stratification.

**Figure 4:**
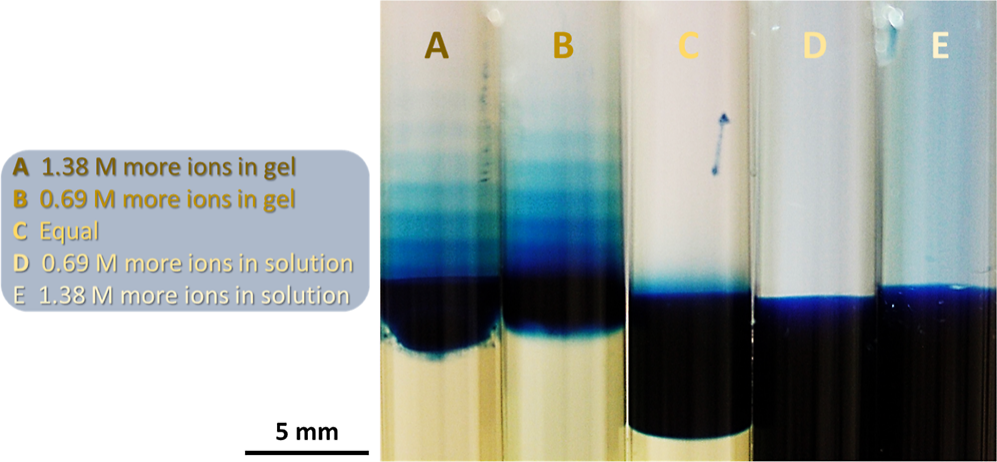
Effect of osmosis concentration on the periodic stratification of the PB colloids in the acid solution above the gel column due to the periodic swelling of the gel at *t* = 168 h. [Fe^3+^] = 0.01 M, [[Fe^II^(CN)_6_]^4–^] = 0.01 M, and [sodium citrate] = 0.3 M in the hydrogel were subjected to 0.6 M HNO_3_ (in 3:1 v/v water/DMF).

To emphasize the importance of gel swelling, we carried out several control experiments in which we varied the osmotic concentration difference between the aqueous phase and the gel (Figure 4 and Movie S3). The pattern was generated only when the osmotic concentration in the gel was greater than that in the aqueous phase. This osmotic stress resulted in the migration of water molecules in the gel, facilitating the swelling of the gel column.^35^ The results of these control experiments proved irrefutably that the osmotic pressure is one of the critical ingredients of the stratification phenomenon. We analyzed the positions of the layers and layer gaps at greater osmotic stress (Figure S4). We found that a higher osmotic concentration difference between the liquid and gel phases resulted in a ∼ 10% increase in both positions of the layers and layer gaps.

A second essential common feature in the systems displaying stratification was the small particle sizes, which prevented the sedimentation of colloids and induced fluid flow due to sedimentation, thus preserving the stable stratification formed. DLS measurement revealed that the average size of PB colloids is ∼400 nm (Figure S5), and the mean zeta potential and conductivity were −1.0 mV and 4.56 mS/cm, respectively (Figure S6), which suggests a highly unstable sample that can aggregate. We primarily used DLS measurements to characterize the size of the colloidal particles instead of SEM and TEM analyses. These analyses require sample preparation and drying on the sample holder, which can cause further particle aggregation, especially in unstable colloidal solutions. Figure S7 shows the SEM micrographs of the PB colloids, indicating the aggregation and formation of larger particles.

Similar stratification structures could be observed in other PBA systems, such as indium(III) and silver(I) PBAs (Figure S8). However, no stratification pattern formed in the liquid phase in the case of cobalt(II) and copper(II) PBAs, which had micron-sized particles (Figure S9 and Movie S4).^25,36^ The formation of smaller colloids can be attributed to the lower values of the solubility products of these PBAs. Iron(III), indium(III) ferrocyanide, and silver(I) ferrocyanide PBAs, producing periodic stratification, have lower solubility products (3.3 × 10^–41^, 1.9 × 10^–44^, and 1.6 × 10^–41^, respectively) compared to those of cobalt(II) ferrocyanide (1.8 × 10^–15^) or copper(II) ferrocyanide (1.3 × 10^–16^) showing no pattern formation in the aqueous phase.^37^

Raman spectroscopy of the formed colloids explicitly showed three distinctive peaks in the 2084 and 2150 cm^–1^ region, characteristic of the cyanide stretching vibrations in PB (Figure S10). The peak at 2150 cm^–1^ corresponds to the ν(CN) stretching vibration’s A_1g_ mode for the [Fe(II), Fe(III)] state, along with its shoulder at 2123 cm^–1^, which belongs to CN^–^. In addition, the peak at 2084 cm^–1^ corresponds to E_g_ mode of the *v*(CN) stretching vibration for the [Fe(II), Fe(III)] state.^38−42^ The other characteristic Raman peak for PB, ascribed to the Fe–C stretching vibrations, should appear near 600 cm^–1^.^42,43^ However, these signals were suppressed by the Raman peaks of nitric acid, and DMF remained in the sample even after excessive washing.^44−46^

PB particles were also characterized by FTIR spectroscopy (Figure S11). The peaks at 2083 cm^–1^ correspond to the cyanide stretching vibration *v*(CN). In the literature, the window 500–600 cm^–1^ is ascribed to Fe–C stretching vibrations and its bending mode in PB.^47^ However, due to the interfering peaks of DMF and nitric acid present in the samples, this part of the spectrum cannot be resolved.^48,49^

SEM–EDX of the samples verified the expected iron presence in addition to a small peak for potassium, which accounts for its intercalation (Figure S12). Charge-balancing intercalated potassium ions make the PB soluble in water. The PB containing potassium ions is the so-called “soluble PB”, which explains the PBs remaining as colloids in the liquid phase.^50,51^

To understand and describe the periodic stratification in the aqueous phase observed in the experiments, we developed a reaction–diffusion–advection model using a kinetic model of the formation of PB by the DR method. The kinetic model comprises four chemical reactions. The precipitation process was described by the following chemical equations with the corresponding chemical rates (*v*) and rate constants (*k*)
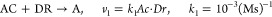
1
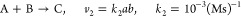
2
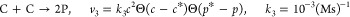
3
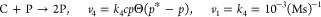
4where *Ac*, *Dr*, *a*, *b*, *c*, and *p* are the concentrations of the chemical species AC (acid—activator), DR (citrate complex—DR), A (metal cation), B (ferrocyanide ion), C (intermediate species of PB), and P (precipitate—PB), respectively. Eq 1 describes the liberation of Fe(III) cations from the Fe(III)-citrate complex induced by a strong acid (in our case, it is nitric acid), which can further react with the ferrocyanide, producing the intermediate species (eq 2). Eq 3 presents the precipitate growth by the aggregation of the intermediate species. Once the precipitate is formed, it can grow by absorbing intermediates into it in threshold-limited processes (eq 4). Θ is the Heaviside step function, and c* and p* are threshold concentrations of precipitation processes described by eqs 3 and 4. The third reaction occurs only when the concentration of the intermediate species reaches c* and the concentration of the precipitate is lower than p*. The heterogeneous reaction (eq 4) takes place only if the concentration of the precipitate is lower than p*. The upward transport due to the periodic swelling of the gel column and sedimentation of colloids were incorporated into the model using an advection term for the precipitate (*p*).

The formation of periodic stratification of PB particles in an aqueous phase can be described in 1D by the following set of partial differential (reaction–diffusion–advection) equations
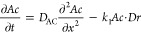
5
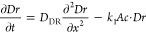
6
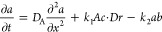
7
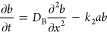
8
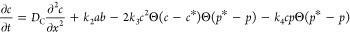
9
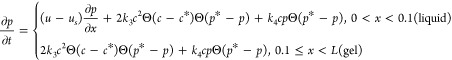
10

We used c* = 0.1 and p* = 3.5 M in the simulations. *L* is the length of the simulation domain. The diffusion coefficient of all species was 10^–9^ m^2^/s except the intermediate species having *D* = 2 × 10^–10^ m^2^/s and precipitate, which did not diffuse. *u* and *u*_s_ are the advection velocities generated by the gel swelling and sedimentation of colloids in the liquid phase, respectively. Two values of the sedimentation velocity were used to test the behavior of the system, *u*_s_ = 6 × 10^–10^ and *u*_s_ = 1.2 × 10^–8^ m/s, corresponding to slow and fast sedimentation of the colloids, respectively. The advection generated by the periodic swelling was implemented in the model using a square wave with a 10^6^ s period and a 1% duty cycle
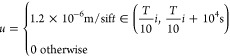
where *i* goes from 0 to 9 and *T* is the length of the simulation (*T* = 10^7^ s). The reaction–diffusion–advection equations (eqs 5–10) were solved with the forward time centered space method using MATLAB software. We applied the following initial conditions to reflect the experimental conditions, namely, the acid (AC) was homogeneously distributed in the liquid phase and the DR and hexacyanoferrate (B) were uniformly distributed in the gel; the liquid phase: *Ac*[*t* = 0, *x* ∈ (0, 0.1)] = 1 M, *Dr*[*t* = 0, *x* ∈ (0, 0.1)] = *a*[*t* = 0, *x* ∈ (0, 0.1)] = *b*[*t* = 0, *x* ∈ (0, 0.1)] = *c*[*t* = 0, *x* ∈ (0, 0.1)] = *p*[*t* = 0, *x* ∈ (0, 0.1)] = 0; gel: *Dr*[*t* = 0, *x* ∈ (0.1, *L*)] = *b*[*t* = 0, *x* ∈ (0.1, *L*)] = 1 M, *Ac*[*t* = 0, *x* ∈ (0.1, *L*)] = *a*[*t* = 0, *x* ∈ (0.1, *L*)] = *c*[*t* = 0, *x* ∈ (0.1, *L*)] = *p*[*t* = 0, *x* ∈ (0.1, *L*)] = 0. We used Dirichlet boundary conditions for all chemical species at *x* = 0, *Ac*(*t*, *x* = 0) = 1.0 M, *Dr*(*t*, *x* = 0) = *a*(*t*, *x* = 0) = *b*(*t*, *x* = 0) = *c*(*t*, *x* = 0) = *p*(*t*, *x* = 0) = 0 and *x* = *L* (at the end of the computational domain), *Dr*(*t*, *x* = *L*) = *b*(*t*, *x* = *L*) = 1 M, *Ac*(*t*, *x* = *L*) = *a*(*t*, *x* = *L*) = *c*(*t*, *x* = *L*) = *p*(*t*, *x* = *L*) = 0, respectively. The length of the simulation domain, grid spacing, and the time step were *L* = 0.2 m, Δ*x* = 2 × 10^–4^ m, and Δ*t* = 10 s, respectively.

**Figure 5:**
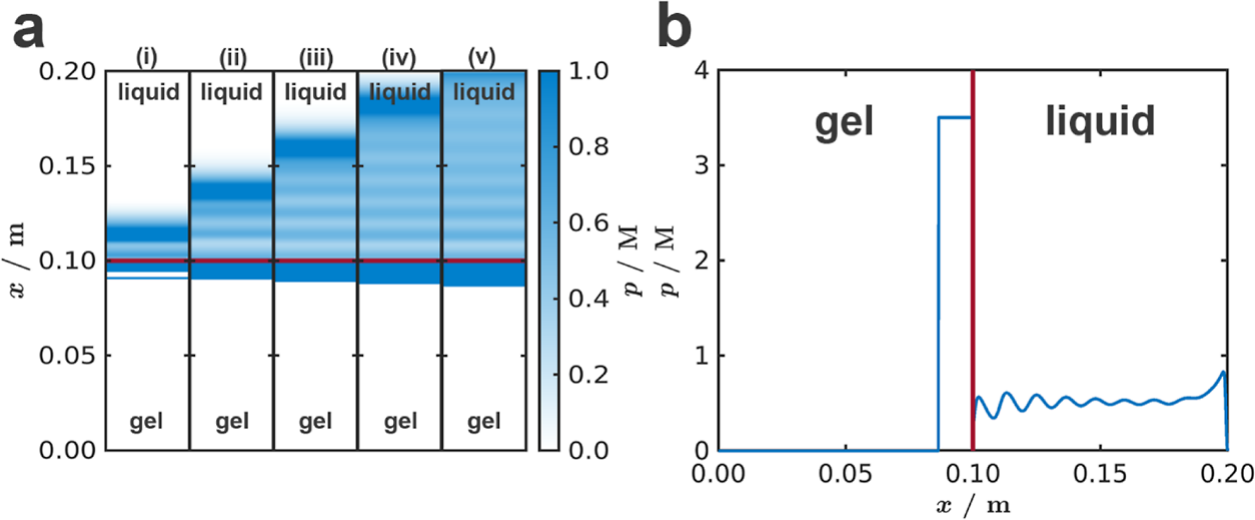
Results of the numerical simulations—periodic stratification of the PB colloids in the liquid phase using *u*_s_ = 6 × 10^–10^ m/s. Concentration distribution of the precipitate (*p*) in the gel and liquid phase, (a) quasi-2D simulation results at various times (i) *t* = *T*/5, (ii) *t* = 2*T*/5, (iii) *t* = 3*T*/5, (iv) *t* = 4*T*/5, and (v) *t* = *T*, where *T* is the simulation time. (b) Cross section of the concentration of the precipitate along the *x*-axis. The gel–liquid interface is at *x* = 0.1 m (indicated by a solid red line).

Figure 5 and Movie S5 present the results of the numerical simulations. An extended reaction–diffusion model with an advection term for the precipitate incorporating the periodic expansion of the gel column (*u*) and sedimentation of particles (*u*_s_) was able to reproduce qualitatively the experimentally observed stratification of PB colloids in the aqueous phase. We have observed in the numerical simulations that the crucial factor in the pattern formation is the ratio of the sedimentation velocity and the upward velocity of the particles due to gel swelling. If the sedimentation velocity is very high, then the pattern could not be evolved. Figure S13 and Movie S6 show this effect, which is in good correspondence with the experiments, where the stratification occurred only in the case of a low solubility of the precipitate (when the particle size and the sedimentation velocity were small).

## Conclusions

In this work, we investigated the formation of a stable periodic stratification of colloidal particles of PB and PBAs in a liquid phase placed on the top of a solid hydrogel column in a test tube. The colloids were generated at the liquid–gel interface using a DR method. Interestingly, the pattern formation did not proceed in the gel, but due to the periodic swelling of the gel driven by the osmotic stress, a stable stratification of colloids appeared in the aqueous phase. This type of pattern differs from the patterns generated in periodic precipitation, in which pattern formation occurs in the gel and the phenomenon is driven by the diffusion front. This study can provide new insights into the design and engineering of periodic structures in a liquid phase by coupling the mechanical properties of the gels to precipitation processes.

## Supporting Information Available

The following files are available free of charge. The Supporting Information is available free of charge at https://pubs.acs.org/doi/10.1021/acs.langmuir.4c00533.

Description of the numerical simulations and calculation of ion concentrations and results of additional experiments and characterization of the pattern and the PB samples (Raman spectroscopy, FTIR, and SEM–EDX) (PDF)
Swelling of the gel column over time and formation of the stratified layers of PB colloids (MP4)
Periodic stratification observed in glass test tubes with various diameters and a plastic test tube (MP4)
Control experiments carried out by varying the osmotic concentration difference between the aqueous phase and the gel (MP4)
Formation of Fe, Cu, and Co PBAs (MP4)
Numerical simulation using low sedimentation velocity of the colloids (MP4)
Numerical simulation using high sedimentation velocity of the colloids (MP4)
