# Supplementary material for: Periodic Stratification of Colloids in a Liquid Phase Produced by a Precipitation Reaction and Gel Swelling
Source: Langmuir. 2024 May 17;40(21):11049–55. doi: 10.1021/acs.langmuir.4c00533 (PMC11140740; doi:10.1021/acs.langmuir.4c00533)
Supplement: Supplementary file 1 — la4c00533_si_001.pdf [file la4c00533_si_001.pdf]

Supporting Information

Periodic Stratification of Colloids in a Liquid Phase Produced by a Precipitation Reaction and Gel Swelling

Pedram Tootoonchian [1], Gábor Holló [2#], Rana Uzunlar [1], Istvan Lagzi [2,3], Bilge Baytekin [1,4]**

[1]Chemistry Department, Bilkent University, Ankara 06800, Turkey

[2]Department of Physics, Institute of Physics, Budapest University of Technology and Economics, Budapest H-1111, Hungary

[3]HU-REN–BME Condensed Matter Physics Research Group, Budapest University of Technology and Economics, Budapest H-1111, Hungary

[4]UNAM National Nanotechnology Research Center, Bilkent University, Ankara 06800, Turkey

Present Address

[#] Department of Fundamental Microbiology, University of Lausanne, Lausanne 1015, Switzerland (G. H.)

Calculation of ion concentration

To prepare the gel, we used 0.01 M of $FeCl_3 \cdot 6H_2O$, 0.01 M of $K_4[Fe^{II}(CN)_6] \cdot 3H_2O$, and 0.3 M of trisodium citrate. Therefore, the concentrations of the ions were the following:

$[Fe^{3+}]$ = 0.01 M

$[Cl^{-}]$ = 0.03 M

$[[Fe^{II}(CN)_6]^{4-}]$ = 0.01 M

$[K^{+}]$ = 0.04 M

$[C_3H_5O(COO)_3^{3-}]$ = 0.3 M

$[Na^{+}]$ = 0.9 M

This will give a total of 1.29 M of ions in the gel medium. After gelation, 0.6 M of HNO_3 was placed on top of the gel. Therefore, 1.29 M – 0.6 M = 0.69 M more ions were present in the gel compared to the aqueous phase.

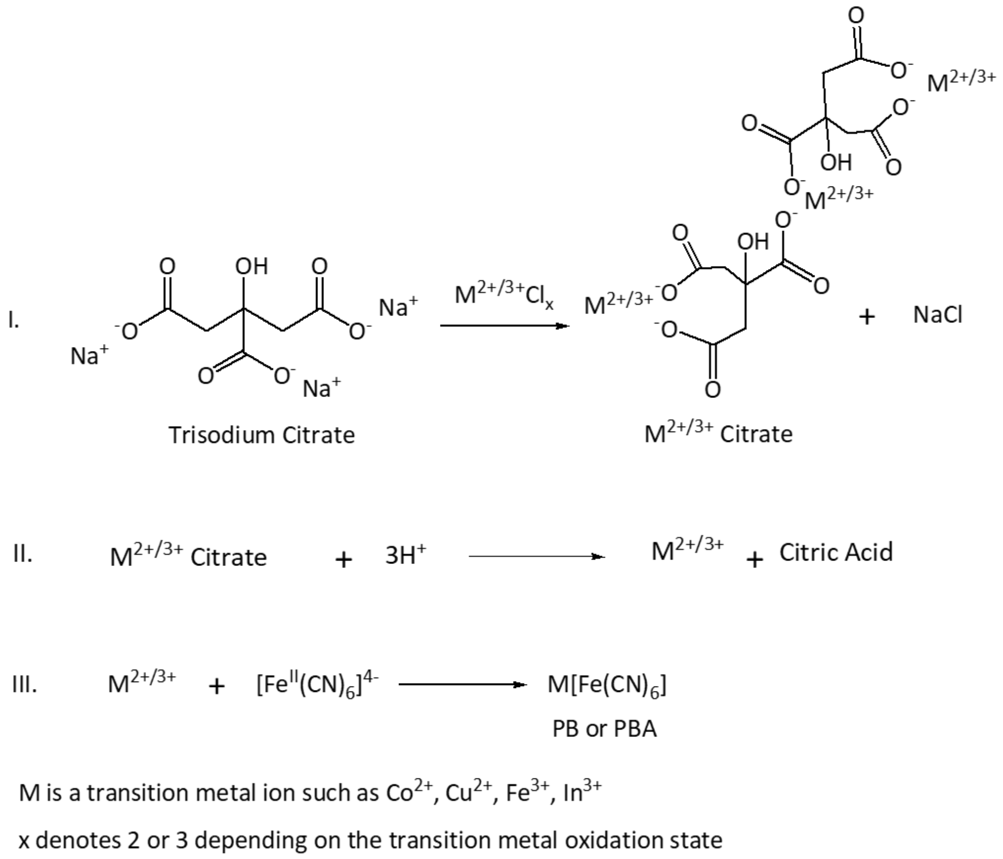

Figure S1. The mechanism of the formation of PB and PBA by dormant reagent method where metal cations and citrate (deactivator) ions bind and form the dormant reagent, which is subsequently released by acid to react with ferrocyanide.

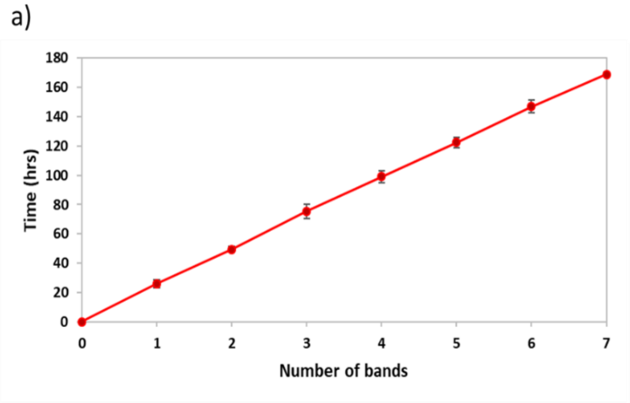

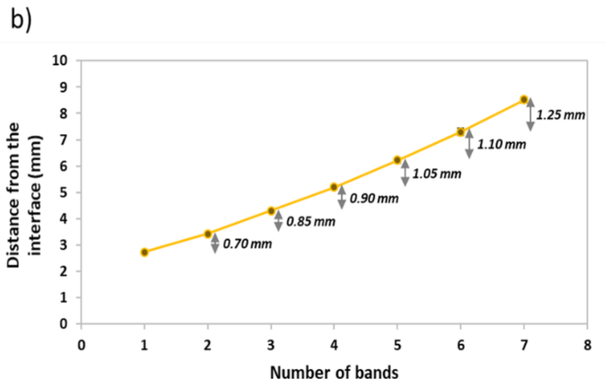

Figure S2. a) Evolution of the stratification of the PB colloids in the acid solution above the gel. b) The position of the colloid layers measured from the liquid-gel interface (Figure 2). The experimental conditions were the following: [Fe^{3+}] = 0.01 M, [[$Fe^{II}(CN)_6$]$^{4-}$] = 0.01 M, and [sodium citrate] = 0.3 M in the hydrogel was subjected to 0.6 M HNO_3 (in 3:1 v:v water/DMF). The grey arrows and corresponding lengths indicate the distance between two consecutive layers of colloids. In the experiment, 0.69 M more ions were in the gel than in the solution phase. The diameter of the glass tube was 5 mm.

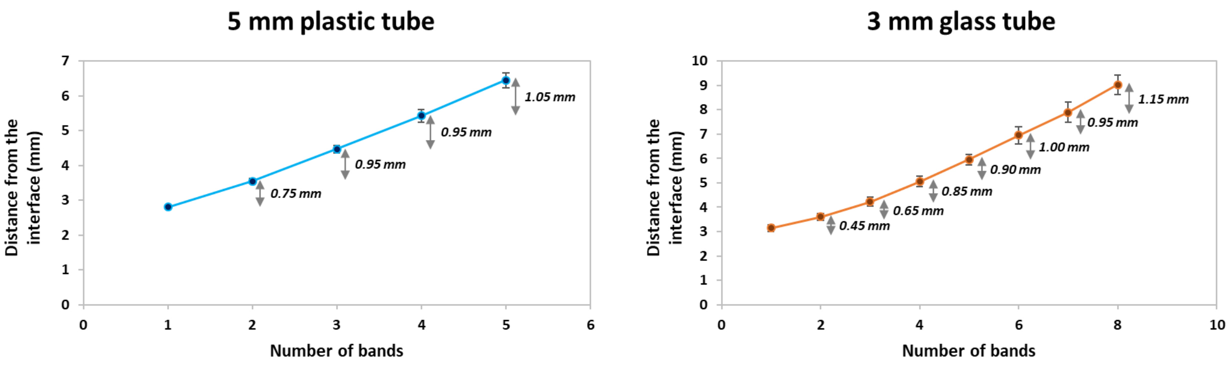

Figure S3. The position of the colloid layers formed is measured from the liquid-gel interface using a plastic tube with a diameter of 5 mm and a glass tube with a diameter of 3 mm (Figure 3 A and B). The experimental conditions were the following: $[Fe^{3+}]$ = 0.01 M, $[[Fe^{II}(CN)_6]^{4-}]$ = 0.01 M, and [sodium citrate] = 0.3 M in the hydrogel was subjected to 0.6 M HNO_3 (in 3:1 v:v water/DMF). The grey arrows and corresponding lengths indicate the distance between two consecutive layers of colloids. In the experiment, 0.69 M more ions were in the gel than in the solution phase.

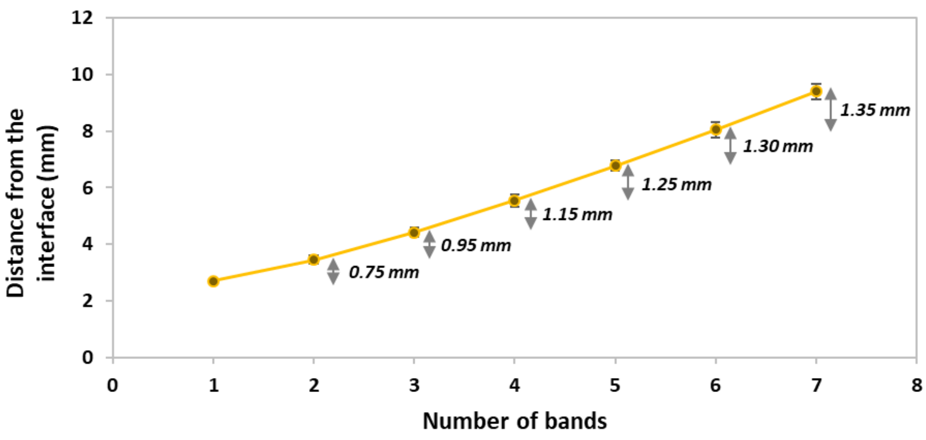

Figure S4. The position of the colloid layers formed is measured from the liquid-gel interface (Figure 4A). The experimental conditions were the following: $[Fe^{3+}]$ = 0.01 M, $[[Fe^{II}(CN)_6]^{4-}]$ = 0.01 M, and [sodium citrate] = 0.3 M in the hydrogel was subjected to 0.6 M HNO_3 (in 3:1 v:v water/DMF). The grey arrows and corresponding lengths indicate the distance between two consecutive layers of colloids. In the experiment, 1.38 M more ions were in the gel than in the solution phase.

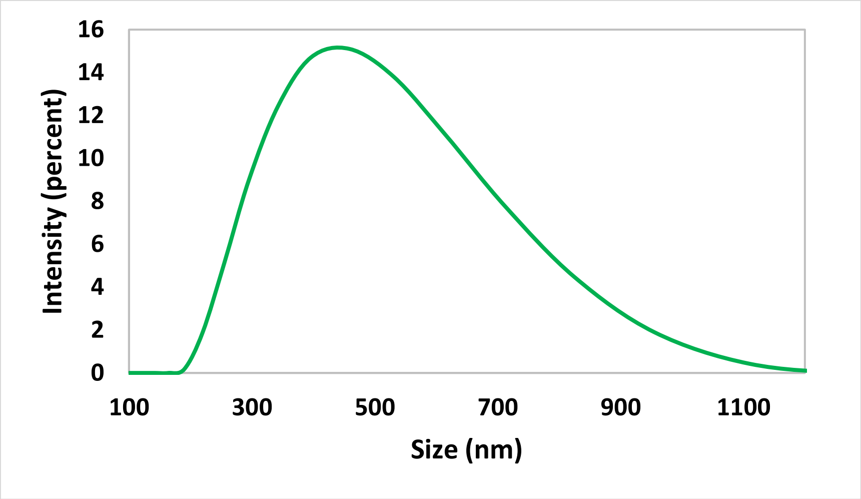

Figure S5. The size distribution of the PB particles measured by dynamic light scattering (DLS) formed in the acid solution at t = 168 h.

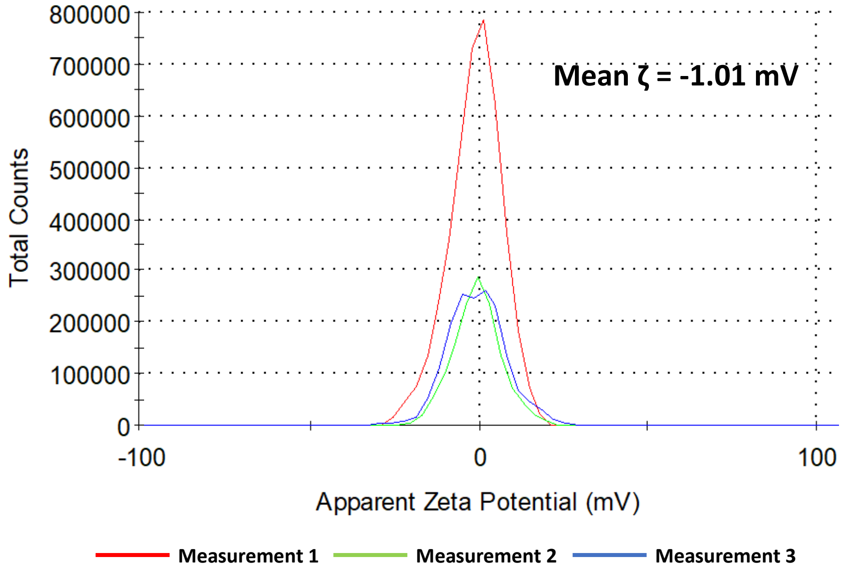

Figure S6. The results of the zeta potential measurements in a sample of PB colloids formed in the acid solution at t = 168 h.

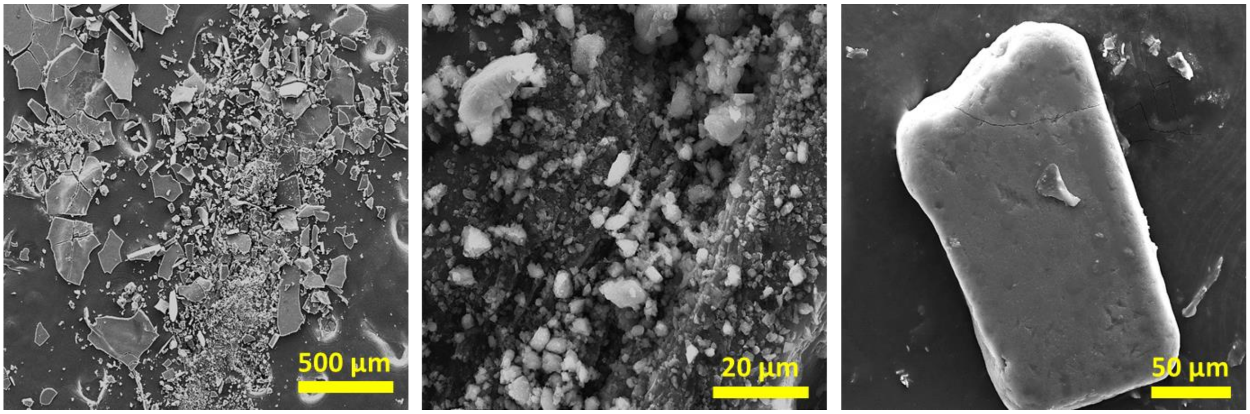

Figure S7. SEM micrographs of the PB colloids formed in the acid solution at t = 168 h.

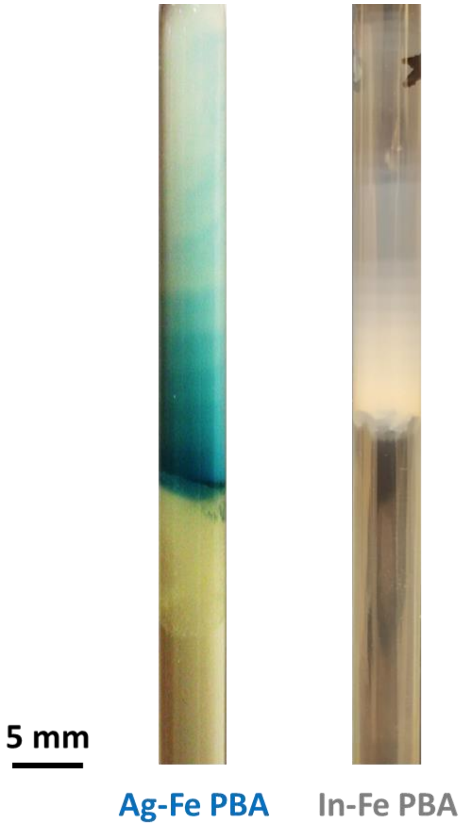

Figure S8. The pattern formation of silver hexacyanoferrate and indium hexacyanoferrate. $[Ag^{+}]$ = 0.01 M, $[[Fe^{II}(CN)_6]^{4-}]$ = 0.01 M, and [sodium citrate] = 0.3 M in the hydrogel is subjected to 0.6 M HNO_3 (in 3:1 water/DMF) for silver hexacyanoferrate precipitation. $[In^{3+}]$ = 0.01 M, $[[Fe^{II}(CN)_6]^{4-}]$ = 0.01 M, and [sodium citrate] = 0.3 M in the hydrogel is subjected to 0.6 M HNO_3 (in 3:1 v:v water/DMF) for indium hexacyanoferrate precipitation.

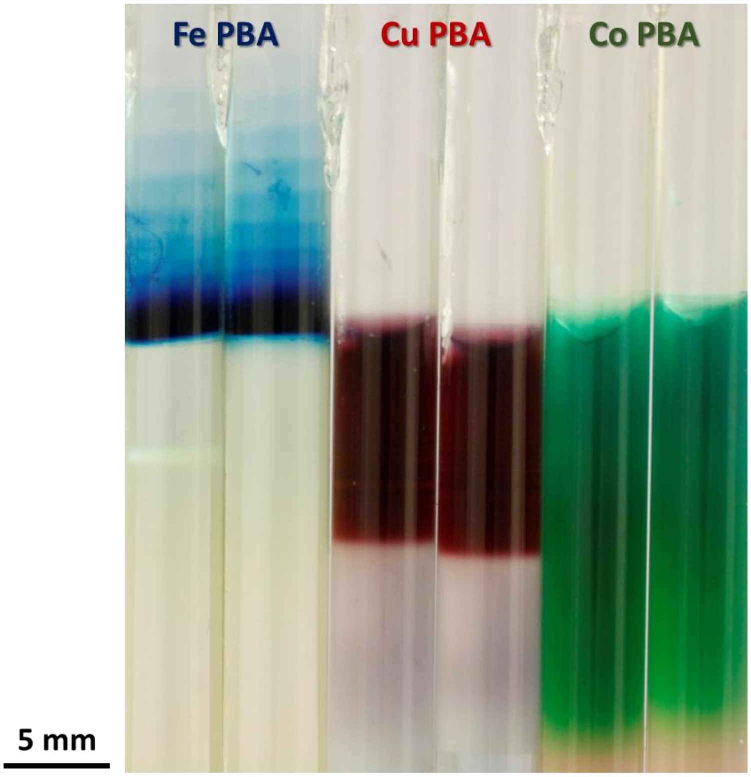

Figure S9. The comparison of diffusion patterns of Fe PBA, Cu PBA, and Co PBA. The Liesegang pattern formation for copper hexacyanoferrate and cobalt hexacyanoferrate requires different concentrations for the cation. Therefore, to have the same osmotic pressure for the comparison of diffusions, we kept the experimental conditions identical. The Fe PBA bands formed after 72 h. For all samples, the cation (Fe^{3+}, Cu^{2+}, Co^{2+}) with 0.01 M concentration and the anion $[[Fe^{II}(CN)_6]^{4-}]$ = 0.01 M in addition to 0.3 M sodium citrate were dissolved in the pre-gel solution, and 0.6 M HNO_3 (in 3:1 water/DMF) was subsequently introduced to the gel from top after gelation.

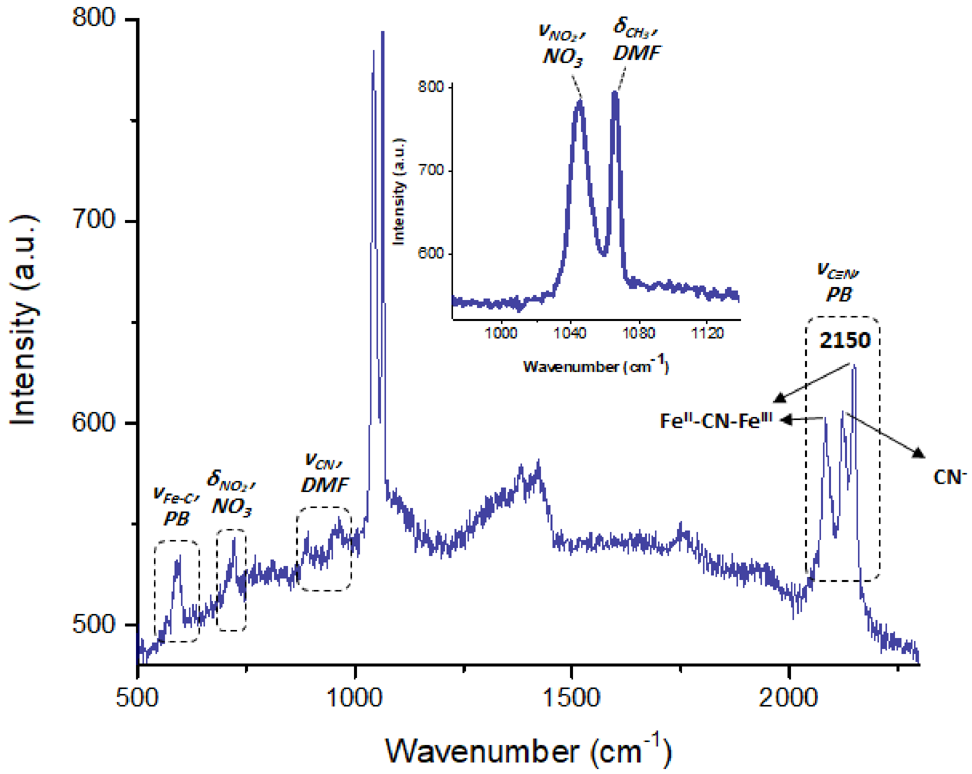

Figure S10. Raman spectrum of the formed PB colloids in the aqueous phase (in 3:1 water/DMF).

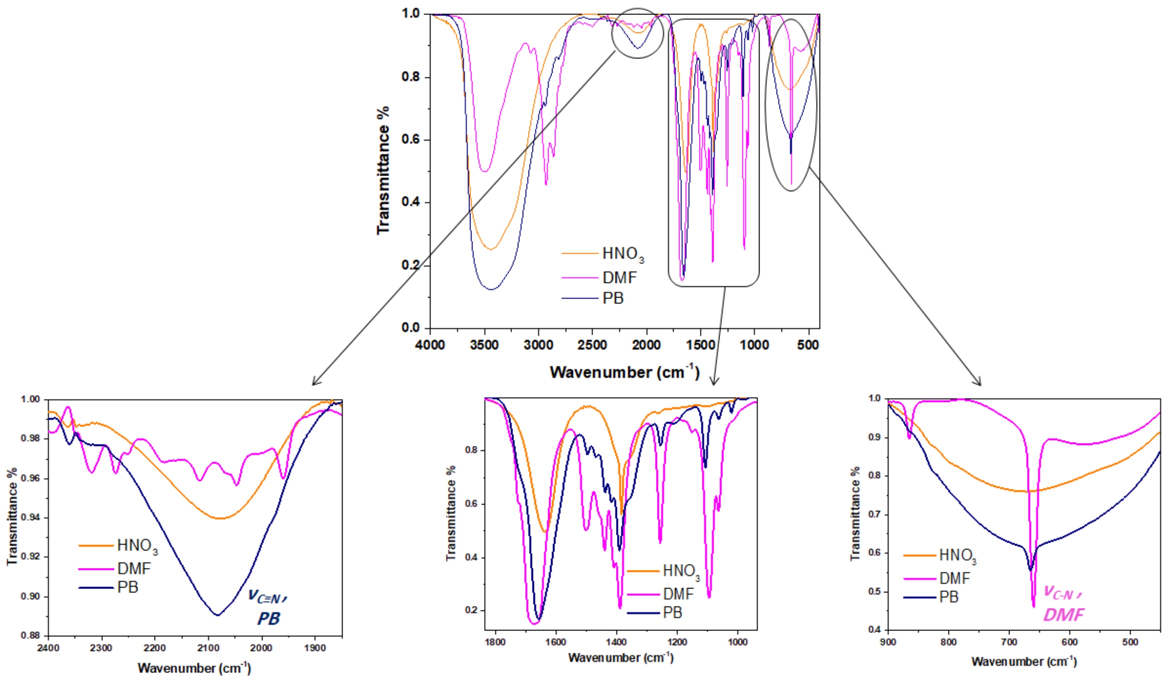

Figure S11. FTIR spectrum of the formed PB colloids in the aqueous phase (in 3:1 water/DMF) in addition to FTIR spectra of HNO_3 and DMF.

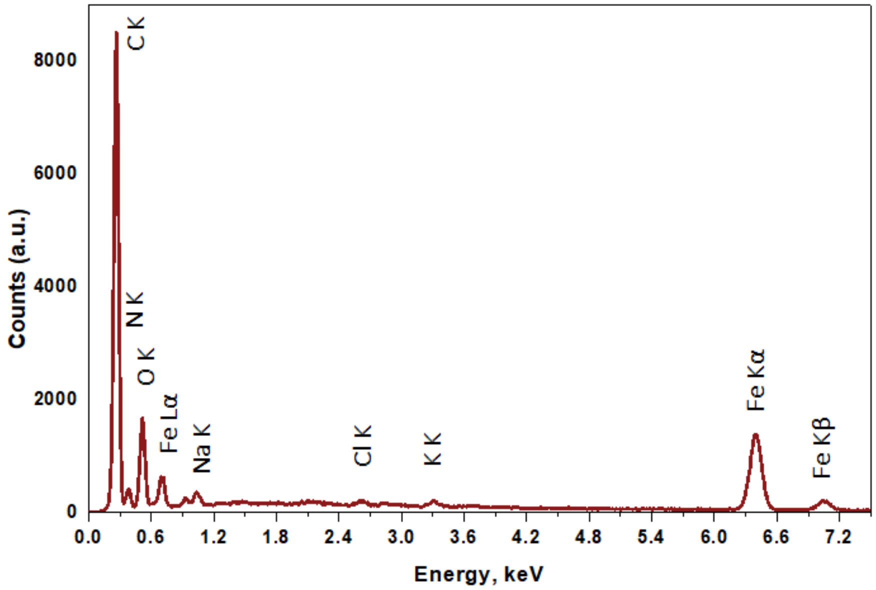

Figure S12. EDX spectrum of the formed PB colloids in the aqueous phase (in 3:1 water/DMF).

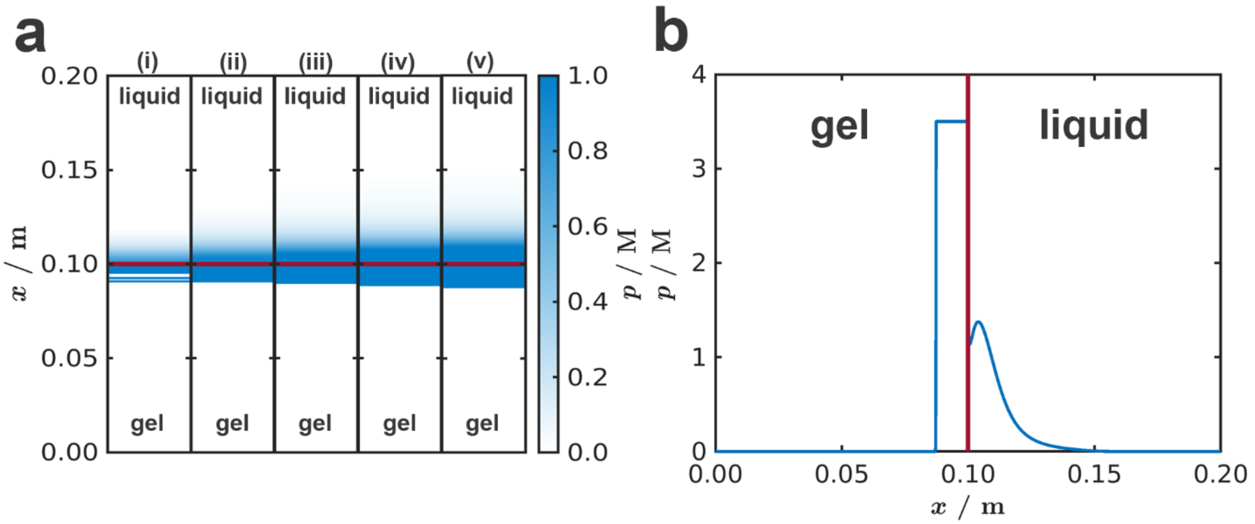

Figure S13. Results of the numerical simulations - distribution of the PB colloids in the liquid phase using u_s = 1.2×10^{-8} m/s (this is 20 times greater than used in Figure 5). Concentration distribution of the precipitate (*p*) in the gel and liquid phase, (a) quasi-2D simulation results at various times (i) $t = T/5$, (ii) $t = 2T/5$, (iii) $t = 3T/5$, (iv) $t = 4T/5$, and (v) $t = T$, where T is the simulation time. (b) cross-section of the concentration of the precipitate along the *x*-axis. The gel-liquid interface is at x = 0.1 m (indicated by a solid red line).
